# Suboptimal adherence to antiretroviral treatment and its predictors among people living with HIV in the era of test and treat

**DOI:** 10.1038/s41598-025-96631-1

**Published:** 2025-04-12

**Authors:** Ismael Ahmed, Fana Tefera, Alemayehu Bekele, Jemal Ayalew, Fasil Tessema, Getinet Abera, Jelaludin Ahmed, Alemayehu Mekonnen, Ashenafi Haile, Fikerte Yohannes, Mirtie Getachew, Saro Abdella, Minesh Shah

**Affiliations:** 1 U.S. Centers for Disease Control and Prevention (CDC), Addis Ababa, Ethiopia; 2 Centre for Innovative Drug Development & Therapeutic Trials for Africa (CDT-Africa), Addis Ababa University, Addis Ababa, Ethiopia; 3 Department of Statistics, College of Natural Science, Wollo University, Dessie, Ethiopia; 4 International Center for AIDS Care and Treatment Programs (ICAP) at Columbia University’s Mailman School of Public Health, Addis Ababa, Ethiopia; 5 Ministry of Health of Ethiopia, Addis Ababa, Ethiopia; 6 Ethiopian Public Health Institute, Addis Ababa, Ethiopia; 7 U.S. Centers for Disease Control and Prevention (CDC), Hanoi, Vietnam

**Keywords:** Antiretroviral therapy, Adherence to ART, HIV, Test and treat, Ethiopia, Africa, Diseases, Infectious diseases, HIV infections, Epidemiology, Infection, Infectious diseases

## Abstract

Despite the success in scaling-up antiretroviral therapy (ART) services in Ethiopia, suboptimal adherence to ART has been an existing challenge. There is a dearth of evidence on the status of adherence to ART following the adoption of test and treat strategy in Ethiopia. This study aimed to investigate on the magnitude of suboptimal adherence and its predictors among patients taking ART. A multicenter prospective cohort study was conducted among adults aged 15 years and above who started ART between March and June 2019 in 39 health facilities (HFs) in Ethiopia. Measurements on sociodemographic, behavioral, and clinical characteristics were taken at baseline and 6- and 12-months following ART initiation. Multivariable logistic regression model using generalized estimating equations was used to identify factors associated with suboptimal adherence. In total, 1229 individuals who started ART were included in the study. The proportion of suboptimal adherence was 8.0% and 7.9% at 6- and 12-months, respectively. Younger age (adjusted odds ratio (AOR) = 2.28 (95% confidence interval (CI) 1.10, 4.74)), being single (AOR = 2.08 (95% CI 1.25, 3.48)), and being a farmer (AOR = 3.21 (95% CI 1.84, 5.61)) were associated with increased risk for suboptimal adherence. Similarly, alcohol intake (AOR = 3.31 (95% CI 2.14, 5.11)), missing clinic appointment (AOR = 5.73 (95% CI 3.76, 8.75)), having opportunistic infections (AOR = 2.86 (95% CI 1.67, 4.88)) and presence of comorbidities (AOR = 3.51 (95% CI 1.89, 6.53)) were associated with higher risk for suboptimal adherence. We observed lower rate of suboptimal adherence to ART following the implementation of test and treat strategy in Ethiopia. Various sociodemographic, clinical, and behavioral factors were found to be independent predictors of suboptimal adherence. The findings highlight the importance of person-centered adherence support based on individual characteristics.

## Introduction

Ethiopia has achieved great success in scaling-up comprehensive human immunodeficiency virus (HIV) care and treatment services, providing life-saving antiretroviral treatment (ART) to about half a million people in partnership with and support from President’s Emergency Plan for AIDS Relief (PEPFAR) and Global Fund in the past two decades^1^. The success of the HIV treatment program depends on the adherence of people living with HIV (PLHIV) to their treatment, as shown by a meta-analysis across > 26 countries that found that optimal adherence was associated with a lower risk of virologic failure^2^. HIV program data in Ethiopia also showed that nonadherence to treatment was the primary factor for most individuals with unsuppressed viral load^3,4^. Optimistically, with enhanced adherence counseling sessions for 3–6 months (which includes baseline individual needs assessment, adherence counselling and education sessions and follow-up telephone calls^5^, most individuals achieved viral re-suppression within few months^6^, providing further evidence that nonadherence was the major factor for unsuppressed viral load. According to the overwhelming body of evidence, PLHIV who adhere to ART and maintain a durable viral load of < 200 copies/mL cannot transmit HIV to sexual partners, a fact driving the message of undetectable equals untransmittable (U = U), which has been used globally to promote adherence and reduce stigma^7^. Conversely, evidence also shows that suboptimal adherence contributes to an increased risk of developing opportunistic infections (OI)^8^ and death^9^.

A systematic review conducted before the era of test and treat strategy in sub-Saharan African countries showed that significant number of PLHIV were nonadherent to medication—with average adherence score of 72.9%^10^. In recent studies conducted in Ethiopia, the level of self-reported good adherence to ART (≥ 95% adherence) varies from region to region. For instance, a study conducted in Benishangul-Gumuz region reported the lowest rate of adherence (60.3%) assessed using visual analogue scale^11^ whereas a study conducted in Southwest region reported the highest rate of adherence (83.3%) assessed using pill count^12^.

Adherence to ART can be affected by various factors related to sociodemographic, clinical, or behavioral characteristics of PLHIV. A meta-analysis conducted before the test and treat era in sub-Saharan African countries reported patient- and provider-related factors that determine nonadherence to ART. The findings showed that being male, use of alcohol, use of traditional or herbal medicine, depression, discrimination and stigmatization, poor social support, and dissatisfaction with healthcare facility and healthcare workers were risk factors for nonadherence to ART^10^.

Though there is evidence on the magnitude and predictors of suboptimal adherence prior to the test and treat era, there are limited studies conducted following the adoption of test and treat strategy in Ethiopia^13,14^. In addition, the available recent studies were limited to a single site, enrolled participants who started ART before and after the implementation of test and treat strategy and had variation in estimating the magnitude of adherence across the geographical regions of Ethiopia^11,12,15,16^. Therefore, this multicenter national study aimed to estimate the magnitude of suboptimal adherence to ART and its predictors among adult PLHIV enrolled in HIV treatment following the adoption of test and treat strategy.

## Methods

### Study design and setting

This was a facility-based prospective cohort study carried out to assess treatment adherence of individuals enrolled in HIV treatment between March and June 2019 and followed for 12 months.

The study was conducted in 39 high caseload public HFs (hospitals and health centers) located in 20 PEPFAR priority towns in Ethiopia during the study period. The towns were distributed in three regions of the country, Amhara, Oromia and Tigray and two city administrations, Addis Ababa and Dire Dawa. These regions include about 45% of the ART cohort of the country. The HFs provide comprehensive care, treatment and support services including HIV counseling and testing, adherence counseling and ART initiation, screening and management of OI, tuberculosis (TB) screening and treatment, case management and laboratory services. Once patients are tested positive at HIV testing points in the facility, they are linked to an ART clinic for confirmatory test and subsequent initiation of ART. Adherence counseling services before and post-ART initiation are provided to clients by clinicians and lay healthcare workers (case managers).

### Study participants

All individuals aged ≥ 15 years who were newly diagnosed with HIV and started ART in the selected HFs during the study period were included in this study. Pregnant women at the time of enrollment into care and treatment were excluded from the study due to a different model of care including frequent follow-up. Those individuals who transferred their ART care from another HFs were excluded due to lack of baseline information.

### Sample size and sampling procedure

This study was part of an ART outcome study that assessed linkage, ART initiation, retention, and viral load suppression along the HIV continuum of care. The detail of the sample size determination has been published elsewhere^17^. In summary, the minimum sample size of the outcome study (N = 1284) was estimated based on the largest sample size required to assess viral suppression at the end of 6-month following ART initiation based on the following parameters: proportion of viral load suppression (65%, program data), α of 0.05, 10% width of confidence interval, and a 1.5 design effect (because sampling was taken over a short period and individuals who arrived outside the enrollment period didn’t have a chance of selection). The estimated sample size was further increased by 20% considering refusal to participate in the study and missing viral load information.

The minimum sample size was proportionally distributed to the 39 study sites based on caseload, clients on ART. Study participant enrollment was started in March 2019 and continued until June 2019 in each site to ensure the required sample size was reached. When fewer study participants than expected were identified and consented in some HFs, the overall sample size for each region was compensated by enrolling more study participants from other high caseload study sites within the region during the data collection period.

### Variables and measurements

Adherence to ART was the outcome of interest for this study which was measured at 6- and 12-months post-ART initiation. Participants were interviewed to assess self-reported adherence using a structured questionnaire at the 6- and 12-months follow-up periods. Questions were adapted from (acquired immunodeficiency syndrome (AIDS) clinical trials group study questionnaire^18^. Participants were asked about the number of missed doses in the last two days and 30 days. Additionally, self-reported adherence was measured by the ART clinician during a patients’ routine follow-up appointments and labeled as “good” adherence if the person missed < 2 doses (≥ 95% adherence), “fair” if the person missed 2–4 doses (85–94% adherence), and “poor” if the person missed ≥ 5 doses (< 85% adherence) out of the 30 doses to be taken during each month of ART follow-up^19^. In this study, individuals who missed < 2 doses (≥ 95% adherence) and ≥ 2 doses (< 95% adherence) in the last 30 days were categorized as having optimal and suboptimal adherence, respectively.

Baseline sociodemographic (age, sex, marital status, educational status, employment status, residence, and income), clinical stage, CD4 count, antiretroviral (ARV) regimen, functional status, disclosure of HIV status, body mass index (BMI), presence of OI, comorbidities (cardiovascular, liver, renal, respiratory, and metabolic diseases), and affective mental health disorders (mainly depression, anxiety and bipolar)), and behavioral (alcohol, khat and tobacco use) variables were considered as time independent variables. Both clinical and behavioral variables that were measured at 6- and 12-months follow-up periods were considered as time varying variables.

The data collection methods were face-to-face interview using local languages in respective regions and medical record review. Data were collected using Open Data Kit (ODK) system, an electronic data collection system, that has two interfaces; ODK collect and ODK aggregate that help to gather data at the field level and central database levels, respectively. The server for the ODK aggregate was physically located at Ethiopian Public Health Association office, Addis Ababa. Data collected at the field level were transferred to the central database at the end of the day of data collection for data completeness review and feedback. A data collection field manual was prepared and used to train data collectors and guide the data collection process. Data collectors were ART prescriber nurses working at the HF. Study team provided on-site support and supervision to ensure data quality.

### Statistical analysis

Data were exported to Stata 16.0 for cleaning, coding, checking for missing values and inconsistencies, and data analysis. Descriptive statistical methods were used to summarize the sociodemographic, clinical, and behavioral characteristics of the study participants. Proportion of individuals with suboptimal adherence was estimated at 6- and 12-months following ART initiation.

A multivariable logistic regression model using generalized estimating equations (GEE) was fit to identify predictors of suboptimal adherence. Variables with missing values > 5% such as CD4 cell count, HIV status disclosure, and income were excluded from the analysis. Additionally, individuals who were transferred out to another facility before 6- or 12-months assessment periods were excluded from the analysis. All variables that had a p-value less than 0.2 in the bivariate analysis were incorporated in the multivariable logistic regression model. Possible interaction of terms between age with tobacco, alcohol and comorbidities were checked for inclusion in the model. For variables with repeated-measures data at baseline, 6- and 12-months (drink alcohol, OIs other than TB or cryptococcal meningitis, and visiting HF based on appointment), we used the baseline data to predict the 6-month outcome and the 6-month data to predict the 12-month outcome, indicating a one-period lag. The final model included all significant variables (p < 0.05) and tested for goodness-of-fit using the Hosmer–Lemeshow test^20^. For this analysis unstructured correlation was used as best working correlation after model comparison based on smallest independence model criterion^21^.

We conducted a sensitivity analysis for the magnitude of adherence by treating individuals who dropped out of the study as having optimal adherence or suboptimal adherence at 6- and 12-months following ART initiation. The sensitivity analysis included additional 428 and 471 study participants at 6- and 12-months, respectively.

### Ethics declaration

The study was approved by the institutional review board of EPHA. This project was reviewed in accordance with Centers for Disease Control and Prevention (CDC) human research protection procedures and was determined to be research, but CDC investigators did not interact with human subjects or have access to identifiable data or specimens for research purposes. Written informed consent was obtained from all the participants and from the legal guardians of the participants who were below 16 years of age. Unique identifiers were assigned to the study participants to keep individual information anonymous. Only data collectors had access to participants’ information. Data collectors signed confidentiality agreements before commencing data collection.

## Results

### Characteristics of study participants

**Fig. 1:**
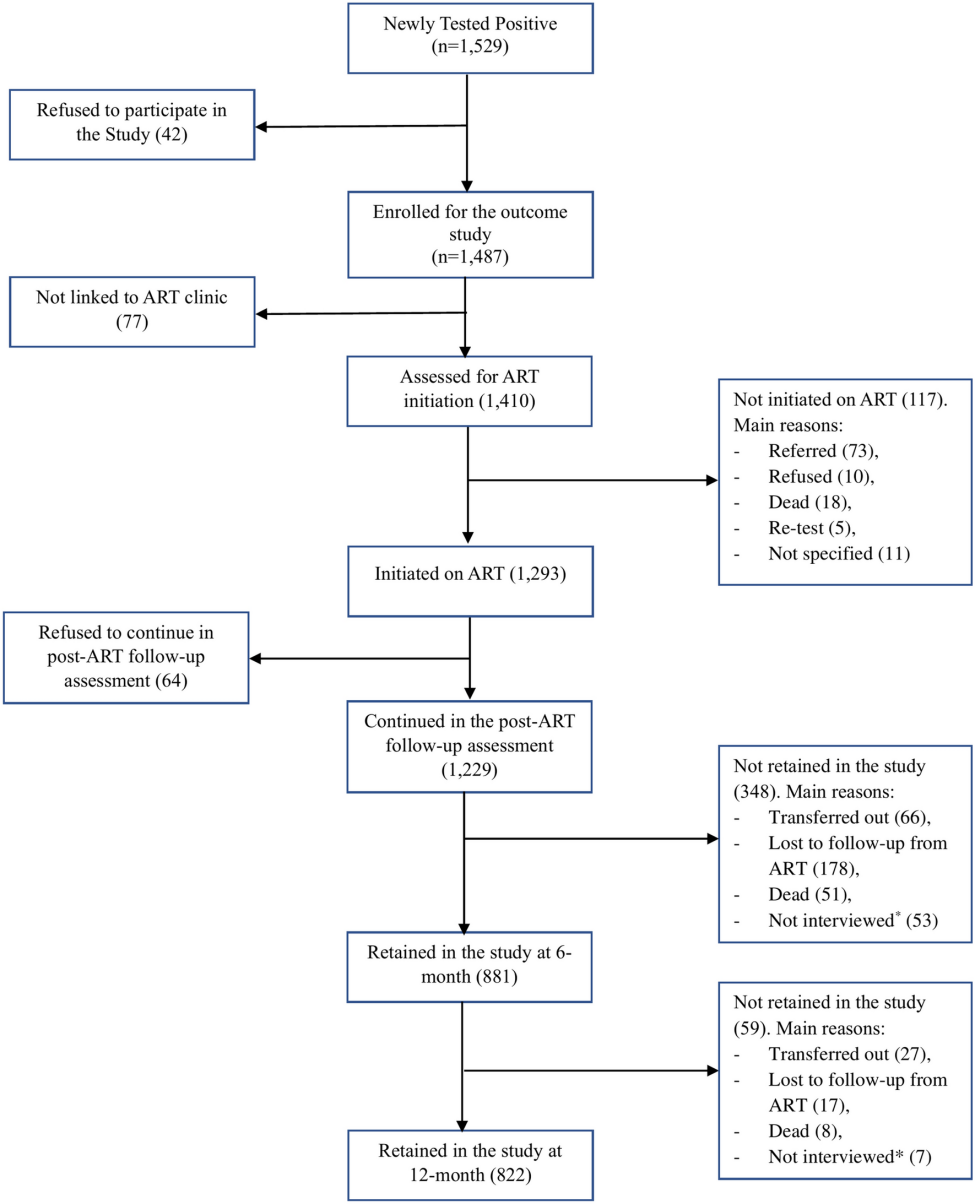
Schematic presentation of study participants enrolment. (*) At sixth and 12th month follow-up, 53 and 7 cases missed interview respectively, but reported to be on ART (active).

Of the 1487 newly tested positive individuals enrolled for the overall outcome study, 1229 individuals who started ART were eligible to be included in this study. Of this, 881 and 822 participants who retained on ART were assessed for treatment adherence at 6- and 12-month, respectively (Fig. 1).

### Baseline sociodemographic characteristics of study participants

About two-thirds (65.8%, n = 808) of study participants were from two regions of Ethiopia, namely Oromia (35.0%, n = 430) and Amhara (30.8%, n = 378) regional states. More than half (54.8%, n = 674) and over a third (35.4%, n = 435) of study participants were women and aged between 25 and 34 years, respectively. The mean age of participants was about 36 years. The majority were married or cohabiting (41.7%, n = 512), employed (44.9%, n = 552), owned mobile phone (80.3%, n = 986), lived in urban area (77.9%, n = 958), and resided in the same district as the HF (69.2%, n = 850) (Table 1).

**Table 1:** Baseline sociodemographic characteristics of PLHIV in Ethiopia, March to June 2019.

Characteristics	Number	Percent
Region (N = 1229)		
Oromia	430	35.0
Amhara	378	30.7
Addis Ababa	248	20.2
Tigray	155	12.6
Diredawa	42	3.4
Residence (N = 1229)		
Urban	958	77.9
Rural	271	22.1
Sex (N = 1229)		
Female	674	54.8
Male	555	45.2
Age group (N = 1229)		
15–24	145	11.8
25–34	435	35.4
35–44	403	32.8
45 +	246	20.0
Mean (SD)	35.6 (10.4)	
Religion (N = 1223)		
Orthodox	931	76.1
Muslim	200	16.4
Protestant	92	7.5
Education (N = 1229)		
No formal school	368	29.9
Primary	424	34.5
Secondary	294	23.9
Higher	143	11.6
Marital status (N = 1229)		
Married/cohabiting	512	41.7
Single	237	19.3
Divorced/separated	342	27.8
Widow	138	11.2
Employment status (N = 1229)		
Employed*	552	44.9
Unemployed	318	25.9
Farmer	143	11.6
Others**	216	17.6
Average monthly household income in ETB (N = 1002)		
< 1800	483	48.2
> 1800	519	51.8
Own mobile phone (N = 1228)		
Yes	986	80.3
No	242	19.7
Patient lives in the same district as the HF (N = 1229)		
Yes	850	69.2
No	379	30.8
Distance to HF (N = 1229)		
< 5 km	602	49.0
5 + Km	627	51.0

*ETB* Ethiopian birr, *HF* health facility, *IQR* interquartile range, *Km* kilometer; *SD* standard deviation. *Government/non-government/self-employed. **Housewife, female sex workers, skilled/unskilled manual, etc. Supplementary information on baseline sociodemographic characteristics of individuals who had adherence measurement at 6- and 12-months compared to those who were dropped out of the study were provided (see Supplementary Table S1 online).

### Baseline and follow-up clinical and behavioral characteristics of study participants

Most of the study participants had normal BMI at baseline and 6th and 12th months follow-up periods. One-third (33.4%, n = 410) of study participants presented with clinical stage III/IV (had advanced HIV disease). Of the total 1229 study participants, only 25.5% (n = 313) had documented baseline CD4 cell count result; of those over half (53%, n = 166) had CD4 cell < 200 count/mm^3^. The proportion of study participants with TB were 9.4, 7.6 and 4.4% at baseline and 6- and 12-months follow-up periods, respectively. The proportion of participants who had mental health disorder (e.g. depression, anxiety and bipolar) was 8.8% (n = 108) at baseline, 4.8% (n = 42) at 6-months, and 3.6% (n = 30) at 12-months. Partner HIV testing was 30.4% (n = 336) at baseline, 47.1% (n = 369) at 6-months, and 52.1% (n = 372) at 12-months (Table 2).

**Table 2:** Baseline and follow-up clinical and behavioral characteristics of PLHIV in Ethiopia, March to June 2019.

Variables	Baseline		6-months		12-months	
	Number	Percent	Number	Percent	Number	Percent
BMI (weight/(height^2^))	(N = 1200)		(N = 881)		(N = 822)	
Underweight	470	39.2	167	19.0	117	14.2
Normal weight	629	52.4	594	67.4	557	67.8
Overweight/obese	101	8.4	120	13.6	148	18.0
Clinical stage	(N = 1229)		(N = 881)		(N = 822)	
Baseline/treatment-stage I	587	47.8	744	84.5	796	96.8
Baseline/treatment-stage II	232	18.9	53	6.0	7	0.9
Baseline/treatment-stage III	317	25.8	66	7.5	14	1.7
Baseline/treatment-stage IV	93	7.6	18	2.0	5	0.6
Baseline CD4 count/mm^3^	(N = 313)					
< 100	87	27.8				
100–199	79	25.2				
200–349	69	22.0				
350–499	32	10.2				
500 +	46	14.7				
Median (IQR)	189 (93, 353)					
Functionality status	(N = 1229)		(N = 881)		(N = 822)	
Working	994	80.9	852	96.7	809	98.4
Ambulatory	203	16.5	28	3.2	13	1.6
Bedridden	32	2.6	1	0.1		
Had TB	(N = 1229)		(N = 881)		(N = 822)	
Yes	115	9.4	67	7.6	36	4.4
No	1114	90.6	814	92.4	786	95.6
Had cryptococcal meningitis	(N = 1096)		(N = 881)		(N = 822)	
Yes	18	1.6	2	0.2	1	0.1
No	1,078	98.4	879	99.8	821	99.9
Had any other OIs	(N = 1096)		(N = 881)		(N = 822)	
Yes	454	41.4	83	9.4	35	4.3
No	642	58.6	798	90.6	787	95.7
Had any comorbidities	(N = 1229)		(N = 881)		(N = 822)	
Yes	86	7.0	35	4.0	22	2.7
No	1,143	93.0	846	96.0	800	97.3
Had any affective mental health disorder	(N = 1229)		(N = 881)		(N = 822)	
Yes	108	8.8	42	4.8	30	3.6
No	1,121	91.2	839	95.2	792	96.4
Time of ART initiation	(N = 1157)					
Same day	596	51.5				
First week (1–7 days)	326	28.2				
Second-fourth week	163	14.1				
After fourth week	72	6.2				
ART regimen	(N = 1229)		(N = 881)		(N = 822)	
EFV-based	749	61.0	397	45.1	193	23.5
DTG-based	455	37.0	459	52.1	608	74.0
Other	25	2.0	25	2.8	21	2.5
Experienced drug side effects			(N = 851)		(N = 812)	
Yes			91	10.7	54	6.7
No			760	89.3	758	93.3
Anyone knows your HIV status	(N = 1073)		(N = 870)		(N = 812)	
Yes	738	68.8	769	88.4	747	92.0
No	335	31.2	101	11.6	65	8.0
Spouse tested for HIV	(N = 977)		(N = 784)		(N = 714)	
Yes	306	31.3	369	47.1	372	52.1
No	337	34.5	191	24.4	150	21.0
IDK	334	34.2	224	28.6	192	26.9
Visited HF on the appointment date			(N = 881)		(N = 822)	
Yes			759	86.2	737	89.7
No			122	13.9	85	10.3
Currently using khat stimulant	(N = 1229)		(N = 881)		(N = 822)	
Yes	130	10.6	43	4.9	33	4.0
No	1,099	89.4	838	95.1	789	96.0
Currently using any form of tobacco	(N = 1229)		(N = 881)		(N = 822)	
Yes	45	3.7	31	3.5	28	3.4
No	1184	96.3	850	96.5	794	96.6
Currently drink alcohol	(N = 1175)		(N = 881)		(N = 822)	
Yes	421	35.8	140	15.9	141	17.2
No	754	64.2	741	84.1	681	82.8

*ART* antiretroviral, *BMI* body mass index, *DTG* Dolutegravir, *EFV* Efavirenz, *HF* health facility, *HIV* human immunodeficiency virus, *IDK* I don’t know, *IQR* interquartile range, *OI* opportunistic infection, *TB* tuberculosis. Supplementary information on baseline clinical and behavioral characteristics of individuals who had adherence measurement at 6- and 12-months compared to those who were dropped out of the study were provided (see Supplementary Table S2 online).

Over half (51.5% n = 596) of study participants started ART on the same day of HIV diagnosis and another 28.2% (n = 326) started ART in the first week (1–7 days) of their diagnosis. The majority (61.0%, n = 749) started an efavirenz (EFV)-based ARV regimen at baseline and were later transitioned to a dolutegravir (DTG)-based regimen at 6-months (52.1%, n = 459) and 12-months (74%, n = 608) follow-up periods. Some study participants reported drug side effects at 6-months (10.7%, n = 91) and 12-months (6.7%, n = 54). The proportion of study participants who were late to their ART pick up appointment dates at 6- and 12-months were 13.9% (n = 122) and 10.3% (n = 85) at 6- and 12-months, respectively (Table 2).

The proportion of study participants who were using khat stimulant were 10.6% (n = 130), 4.9% (n = 43) and 4.0% (n = 33) at baseline and 6- and 12-months follow-up periods, respectively. Over a third (35.8%, n = 421) of participants reported they were using alcohol at baseline, decreased to 15.9% (n = 140) and 17.2% (n = 141) at 6- and 12-months follow-up periods, respectively (Table 2).

### Magnitude of suboptimal ART adherence

Of the study participants who took part in the 6- and 12-months of follow-up assessments, 7.1% (n = 57) and 5.8% (n = 45) reported missing one or more pills in last two days preceding the interview; and 14.5% (n = 116) and 13.3% (n = 101) reported missing one or more pills in one-month time preceding the interview, respectively. The magnitude of suboptimal adherence (missing two or more pills in the last month) was 8.0% (n = 64) and 7.9% (n = 60) at 6- and 12-months, respectively. According to clinicians’ records, the proportion of suboptimal adherence (who had fair or poor adherence) was 6.7% and 7.2% at 6- and 12-months ART follow-up, respectively (Table 3).

**Table 3:** Adherence to ART at 6- and 12-months follow-up among PLHIV in Ethiopia, March to June 2019.

Variables	6-months		12-months	
	Number	Percent	Number	Percent
Number of ART pills missed during the last 2 days*	(N = 801)		(N = 774)	
0	744	92.9	729	94.2
1	52	6.5	40	5.2
2	5	0.6	5	0.6
Number of ART pills missed during the last one month*	(N = 801)		(N = 758)	
0	685	85.5	657	86.7
1	52	6.5	41	5.4
2	32	4.0	25	3.3
> 3	32	4.0	35	4.6
Adherence level based on clinicians’ record**	(N = 857)		(N = 762)	
Good	800	93.3	707	92.8
Fair	34	4.0	38	5.0
Poor	23	2.7	17	2.2

*Adherence measurement based on self-reported missed pills (interview). **Adherence measurement based on clinicians’ record review. *ART* antiretroviral therapy.

### Sensitivity analysis

In a sensitivity analysis that included those who dropped out of the study as having optimal adherence, the magnitude of suboptimal adherence (missing two or more pills in the last month) was 5.2% (n = 64) and 4.9% (n = 60) at 6- and 12-months, respectively (see Supplementary Table S3 online). In contrary, in the analysis that included those who dropped out of the study as having suboptimal adherence, the magnitude of suboptimal adherence was 40.0% (n = 492) and 43.2% (n = 531) at 6- and 12-months, respectively (see Supplementary Table S4 online).

### Predictors of suboptimal adherence to ART

A total of 862 individuals who had adherence measurement at 6-months and/or 12-months were included in the regression model. In multivariable analysis, nine variables were found to be significantly associated with suboptimal adherence to ART. Younger age group (15–24 years) had 2.28 higher odds of having suboptimal adherence compared to older age group (adjusted odds ratio (AOR) = 2.28 (95% confidence interval (CI) 1.10, 4.74)). The odds of having suboptimal adherence were 2.08 (95% CI 1.25, 3.48) times higher among individuals who had never been married compared to married/cohabited individuals (Table 4).

**Table 4:** Factors associated with suboptimal adherence among PLHIV in Ethiopia, March to June 2019.

Variables	COR (95% CI)	AOR (95% CI)	p-value
Age group			
45 +	1.00	1.00	
15–24	2.52 (1.30, 4.87)	2.28 (1.10, 4.74)	0.027
25–34	1.68 (0.97, 2.91)	1.67 (0.94, 2.99)	0.082
35–44	1.18 (0.66, 2.10)	1.27 (0.71, 2.30)	0.423
Marital status			
Married/cohabiting	1.00	1.00	
Single	2.02 (1.25, 3.24)	2.08 (1.25, 3.48)	0.005
Divorced/separated	1.07 (0.65, 1.75)	1.07 (0.65, 1.75)	0.789
Widowed/er	1.25 (0.68, 2.31)	1.69 (0.90, 3.16)	0.100
Employment			
Employed*	1.00	1.00	
Unemployed	1.59 (0.98, 2.60)	1.51 (0.91, 2.50)	0.110
Farmer	3.13 (1.85, 5.31)	3.21 (1.84, 5.61)	< 0.001
Others**	2.48 (1.50, 4.10)	1.81 (1.06, 3.08)	0.030
Currently drink alcohol			
No	1.00	1.00	
Yes	2.59 (1.77, 3.79)	3.31 (2.14, 5.11)	< 0.001
OIs other than TB or cryptococcal meningitis at follow up			
No	1.00	1.00	
Yes	2.31 (1.41, 3.80)	2.86 (1.67, 4.88)	< 0.001
Comorbidities			
No	1.00	1.00	
Yes	2.35 (1.30, 4.22)	3.51 (1.89, 56.53)	< 0.001
BMI			
Under weight	1.00	1.00	
Normal weight	0.65 (0.44, 0.96)	0.93 (0.59, 1.47)	0.762
Overweight/obese	0.19 (0.09, 0.41)	0.37 (0.16, 0.87)	0.023
Visited HF based on appointment			
Yes	1.00	1.00	
No	4.94 (3.37, 7.24)	5.73 (3.76, 8.75)	< 0.001
Constant		0.01 (0.01, 0.03)	< 0.001

*Government/non-government/self-employed. **Housewife, female sex workers, skilled/unskilled manual, etc. *AOR* adjusted odds ratio, *BMI* body mass index, *COR* crude odds ratio, *HF* health facility, *OI* opportunistic infection, *TB* tuberculosis.

Compared to individuals who were employed, the odds of having suboptimal adherence were 3.21 (95% CI 1.84, 5.61) and 1.81 (95% CI 1.06, 3.08) for farmers and individual in “other” group of employment status, respectively. Individuals who drunk alcohol had 3.31 times higher odds of having suboptimal adherence compared to those who did not drink alcohol (AOR = 3.31 (95% CI 2.14, 5.11)) (Table 4).

The odds of having suboptimal adherence were 2.86 for individuals who had an OI (other than TB or Cryptococcal meningitis) during ART follow-up compared to those who did not have OI (AOR = 2.86 (95% CI 1.67, 4.88)). Similarly, individuals who had comorbidities at baseline, had 3.51 higher odds of having suboptimal adherence compared to those who did not have comorbidities (AOR = 3.51 (95% CI 1.89, 6.53). In addition, overweight or obese individuals had 63% reduced odds of having suboptimal adherence during ART follow-up period compared to those who were underweight (AOR = 0.37 (95% CI 0.16, 0.87)). Finally, those individuals who were late to their follow-up appointment date had substantially increased odds of having suboptimal adherence compared to those who visited the HF on the date of their appointment (AOR = 5.73 (95% CI 3.76, 8.75) (Table 4).

## Discussion

This study examined the magnitude of self-reported suboptimal adherence to ART and factors associated with nonadherence including sociodemographic, clinical, and behavioral factors among adult PLHIV enrolled in HIV treatment during the era of test and treat in Ethiopia. Our findings showed that 8.0% of people taking ART had suboptimal adherence both at 6- and 12-months post ART initiation. The study also demonstrated that younger age, being single, being farmer or having other types of employment status (e.g. female sex worker, skilled/unskilled manual), drinking alcohol, missing clinic appointment, and having OIs, comorbidities, and being underweight were associated significantly with increased odds of having suboptimal adherence.

Unlike the findings of limited studies conducted in Ethiopia^13,14,22^, our study finding showed lower suboptimal adherence to ART following test and treatment implementation. In addition, our result is lower than recent cross sectional study findings that enrolled individuals who were started on ART before or after implementation of test and treat strategy. These studies reported suboptimal adherence to ART that ranges from 16.7^12^ to 39.7%^11^ with regional variations. Similarly, a recent systematic review and meta-analysis reported a 20.7% pooled prevalence of nonadherence in Ethiopia^23^. These variations could be mainly due to methodological differences including study design, measurement of adherence, and sample size.

We found several sociodemographic, clinical, and behavioral factors associated with suboptimal adherence to ART in the era of test and treat strategy implementation. Similar with studies conducted in Southwestern^12^, Northwestern^11^ and Northern^24^ part of Ethiopia, younger adults had an increased risk of having suboptimal adherence than their older counterparts. A prior systematic review with meta-analysis also demonstrated that older age has reduced risk for nonadherence^25^. Part of the reason could be younger adults may have less experience in taking medications compared to older adults. Recent qualitative studies conducted in Namibia^26^ and South Africa^27^ revealed that lack of disclosure, lack of awareness about the importance of consistent medication, inadequate family support, and economic problems were common risk factors that affect adherence among young adults. Additionally, younger adults may engage in risky behaviors such as use of drugs and alcohol^28,29^ that could affect their treatment adherence.

Unlike the findings of prior studies, this study identified an association between marital status and suboptimal adherence. We found that single individuals were at higher risk of having suboptimal adherence compared to those who were married or cohabited. This could be explained by the importance of family support in the HIV care^15,30^. In support of this argument, a study conducted in Southwest Ethiopia demonstrated that married individuals had lower risk of dropout from HIV care compared to single or divorced counterparts^31^. In relation to employment status, we found that farmers or those with “other” employment status (e.g. housewife, female sex workers, skilled/unskilled manual) were more likely to have suboptimal adherence compared to employed individuals. Though we have not come across with studies that support this finding, a study conducted in Southwest Ethiopia reported that being famer was a risk factor for dropout from HIV care^31^ which has direct correlation with nonadherence to treatment. Furthermore, the type of work an individual engaged in could be a risk factor for suboptimal treatment adherence. For instance, female sex workers are more vulnerable to other risky behaviors including use of drugs and alcohol that could affect the likelihood of taking ARVs. The higher level of HIV drug resistance and virologic failure among female sex workers in Ethiopia^32^ supports this argument. Further studies are needed to investigate factors affecting adherence to ART among these group of population.

Consistent with several earlier studies conducted in developing countries such as Ethiopia^16,33^, Nigeria^34^, South Africa^35^, Uganda^36,37^, India^38^, and Nepal^39^, the present study demonstrated an association between drinking alcohol and suboptimal adherence. Alcohol influence treatment adherence behavior through the cognitive effects of alcohol (leading to unintentional ART nonadherence) or medication-related beliefs about mixing alcohol and ART (leading to intentional ART nonadherence)^40^. Furthermore, evidence shows that the effect of alcohol in HIV prevention and treatment is complex. For instance, alcohol drinkers, compared to those who abstain, are more likely to engage in high-risk sexual behavior^41^. Consequently, they may acquire HIV, or they may be a potential source to transmit drug resistance virus due to nonadherence and lack of viral suppression. Evidence also shows that alcohol use among PLHIV is a common problem in Ethiopia. According to a systematic review and meta-analysis conducted in Ethiopia, the pooled prevalence of lifetime, current and hazardous alcohol use among HIV patients were 36.4%, 19.0% and 21.6%, respectively^42^. These findings point to the importance of integrating evidence-based interventions into HIV care to support PLHIV who use, or abuse alcohol to improve adherence to treatment and attain HIV epidemic control in the country.

Similar to previous studies in Ethiopia, our study showed an association between suboptimal adherence and having OIs^11^ and comorbidities^12,15,23,43^. The relationship between developing OIs or comorbidities and suboptimal adherence could be bidirectional. For instance, patients who have OI could have difficulty of taking/swallowing ARVs and/or poor drug absorption due to gastrointestinal problems, or they may skip ARV doses due to pill burden^44^. On the other hand, suboptimal adherence could result in uncontrolled replication of the virus which deplete the immunity of the patient, leading to development of OIs^45^. Similarly, our study revealed an association between BMI and adherence to ART which could be because of the effect of malnutrition on missing clinical appointment and not taking ARVs regularly^46^. In support of this argument, evidence in Ethiopia shows food insecurity and malnutrition were more frequent in individuals with nonadherence^47^. In general, the available evidence indicates the need for strengthening early detection and management of OIs, comorbidities and malnutrition.

Another predictor of suboptimal adherence identified in this study was missing clinical appointment date. The finding was supported by a study conducted in Uganda that highlighted an association between missing doses and missing appointments^37^. Additionally, a recent explorative qualitative study from South Africa revealed that those individuals missing clinic appointment were likely to interrupt HIV care^48^. Indeed, those who missed clinic appointment may run out of ARV supply and discontinue treatment.

Our study has some limitations. Firstly, the study assessed adherence to ART for individuals who retained in care at 6- and 12-months following ART initiation—excluding those who dropped off for various reasons. For instance, 195 individuals who were lost to follow-up are potentially nonadherent to ART. In addition, over 90 individuals who transferred their care to another HFs during the follow-up period were dropped out of the study due to difficulty of measuring their adherence. Therefore, our suboptimal adherence could be underestimated, and caution should be taken while interpreting our results. Secondly, due to the nature of self-reported medication adherence measurement^49^, our suboptimal adherence estimate could be affected by social desirability bias. However, a study conducted to evaluate the validity of self-reported medication adherence compared with electronically monitored adherence and plasma viral load from eight United States multisite adherence studies demonstrated no evidence of greater overestimation of self-reported adherence^50^. Additionally, efforts were made to maximize the validity of the assessment while constructing the questions and emphasizing the aim of the study and confidentiality of the responses during interviews. Thirdly, our study was limited to secondary data for CD4 cell count. We have excluded this variable from the analysis due to significant proportion of missing data in the medical records. This was due to interruption of CD4 cell count test in most of the study sites. However, evidence from a South African study that showed no association between CD4 cell count and adherence to ART during test and treat^51^, suggests that excluding CD4 cell count from our analysis may not have a meaningful effect on study results.

Despite the limitations, our study has some strengths. Unlike most of the prior studies conducted in Ethiopia that were dependent on secondary data, our study managed to measure some important behavioral and clinical variables including substance use and mental health status which are key in determining adherence to ART. Our study employed advanced statistical model (GEE model) which is appropriate to analyze repeated measures. Additionally, our study was a multicenter study conducted in 39 high caseload HFs in five major regional administrations of the country which can represent PLHIV at national level.

## Conclusion

In summary, our study revealed several sociodemographic, behavioral, and clinical risk factors that predict the level of adherence to ART. Targeted and person-centered adherence interventions needs to be strengthened based on individual characteristics. Additionally, integrating evidence-based interventions (e.g. U = U messaging) into counseling services would further encourage PLHIV to attain optimal adherence to their medication, and improve HIV treatment outcomes.

## Supplementary Information

Supplementary Information.

## Data Availability

The datasets used and/or analyzed during the current study are available from the corresponding author on reasonable request.
