# Supplementary material for: Suboptimal adherence to antiretroviral treatment and its predictors among people living with HIV in the era of test and treat
Source: Sci Rep. 2025 Apr 12;15:12666. doi: 10.1038/s41598-025-96631-1 (PMC11993605; doi:10.1038/s41598-025-96631-1)
Supplement: Supplementary file 1 — Supplementary Information. [file 41598_2025_96631_MOESM1_ESM.pdf]

**Suboptimal adherence to antiretroviral treatment and its predictors among people living with HIV in the era of test and treat**

*Ismael Ahmed, Fana Tefera, Alemayehu Bekele, Jemal Ayalew, Fasil Tessema, Getinet Abera, Jelaludin Ahmed, Alemayehu Mekonnen, Ashenafi Haile, Fikerte Yohannes, Mirtie Getachew, Saro Abdella, Minesh Shah*

Supplementary Table S1: Baseline sociodemographic characteristics of PLHIV in Ethiopia, March to June 2019

| **Characteristics** | **Participants assessed at least once for adherence*** | | **P-value** |
|---|---|---|---|
| | **No** | **Yes** | |
| Region (N=1,229) | | | <0.001 |
| Amara | 79 (21.5%) | 299 (34.7%) | |
| Oromia | 159 (43.3%) | 271 (31.4%) | |
| Addis Ababa | 65 (17.7%) | 163 (18.9%) | |
| Tigray | 43 (11.7%) | 112 (13.0%) | |
| Diredawa | 21 (5.7%) | 17 (2.0%) | |
| Residence (N=1,229) | | | 0.872 |
| Urban | 285 (77.7%) | 673 (78.1%) | |
| Rural | 82 (22.3%) | 189 (21.9%) | |
| Sex (N=1,229) | | | 0.124 |
| Female | 189 (51.5%) | 485 (56.3%) | |
| Male | 178 (48.5%) | 377 (43.7%) | |
| Age group (N=1,229) | | | 0.007 |
| 15 - 24 | 57 (15.5%) | 88 (10.2%) | |
| 25 - 34 | 136 (37.1%) | 299 (34.7%) | |
| 35 - 44 | 117 (31.9%) | 286 (33.2%) | |
| 45+ | 57 (15.5%) | 189 (21.9%) | |
| Religion (N=1,223) | | | 0.054 |
| Othodox | 275 (75.3%) | 656 (76.5%) | |
| Muslim | 53 (14.5%) | 147 (17.1%) | |
| Protestant | 37 (10.1%) | 55 (6.4%) | |
| Education (N=1,229) | | | 0.082 |
| No formal school | 116 (31.6%) | 252 (29.2%) | |
| Primary | 135 (36.8%) | 289 (33.5%) | |
| Secondary | 86 (23.4%) | 208 (24.1%) | |
| Higher | 30 (8.2%) | 113 (13.1%) | |
| Marital status (N=1,229) | | | 0.092 |
| Married/cohabiting | 142 (38.7%) | 370 (42.9%) | |
| Single | 79 (21.5%) | 158 (18.3%) | |
| Divorced/Separated | 113 (30.8%) | 229 (26.6%) | |
| Widowed | 33 (9.0%) | 105 (12.2%) | |

| | | | |
|---|---|---|---|
| Employment status (N=1,229) | | | 0.028 |
| Employed** | 144 (39.2%) | 408 (47.3%) | |
| Unemployed | 104 (28.3%) | 214 (24.8%) | |
| Farmer | 41 (11.2%) | 102 (11.8%) | |
| Others*** | 78 (21.3%) | 138 (16.0%) | |
| Average monthly household income in ETB (N=1,002) | | | <0.001 |
| < 1800 | 185 (62.1%) | 298 (42.3%) | |
| 1800+ | 113 (37.9%) | 406 (57.7%) | |
| Has mobile phone (N=1,228) | | | <0.001 |
| Yes | 270 (73.6%) | 716 (83.2%) | |
| No | 97 (26.4%) | 145 (16.8%) | |
| Patient lives in the same city as the HF (N=1,229) | | | 0.515 |
| Yes | 249 (67.8%) | 601 (69.7%) | |
| No | 118 (32.2%) | 261 (30.3%) | |
| Distance to HF (N=1,229) | | | 0.202 |
| < 5 km | 190 (51.8%) | 412 (47.8%) | |
| 5+ km | 177 (48.2%) | 450 (52.2%) | |

*Had adherence assessment at 6- and/or 12-months. **Government/non-government/self-employed. ***Housewife, female sex workers, skilled/unskilled manual, etc.

**Abbreviations**: ETB, Ethiopian birr; HF, health facility; IQR, interquartile range; Km, kilometer; SD, standard deviation.

Supplementary Table S2: Baseline clinical and behavioral characteristics of PLHIV in Ethiopia, March to June 2019

| **Characteristics** | **Participants assessed at least once for adherence*** | | **P-value** |
|---|---|---|---|
| | **No** | **Yes** | |
| BMI (N=1,200) | | | 0.047 |
| Underweight | 150 (42.9%) | 320 (37.6%) | |
| Normal weight | 180 (51.4%) | 449 (52.8%) | |
| Overweight/Obese | 20 (5.7%) | 81 (9.5%) | |
| Clinical stage | | | 0.054 |
| Stage I | 165 (45.0%) | 422 (49.0%) | |
| Stage II | 63 (17.2%) | 169 (19.6%) | |
| Stage III | 101 (27.5%) | 216 (25.1%) | |
| Stage IV | 38 (10.4%) | 55 (6.4%) | |
| Baseline CD4 count/mm$^3$ (N=313) | | | 0.126 |
| < 100 | 30 (34.5%) | 57 (25.2%) | |
| 100-199 | 25 (28.7%) | 54 (23.9%) | |
| 200-349 | 18 (20.7%) | 51 (22.6%) | |
| 350-499 | 4 (4.6%) | 28 (12.4%) | |
| 500+ | 10 (11.5%) | 36 (15.9%) | |
| Functional status (N=1,229) | | | 0.003 |

| | | | |
|---|---|---|---|
| Working | 278 (75.7%) | 716 (83.1%) | |
| Ambulatory | 73 (19.9%) | 130 (15.1%) | |
| Bedridden | 16 (4.4%) | 16 (1.9%) | |
| Had TB (N=1,229) | | | 0.003 |
| No | 319 (86.9%) | 795 (92.2%) | |
| Yes | 48 (13.1%) | 67 (7.8%) | |
| Had cryptococcal meningitis (N=1,096) | | | 0.724 |
| No | 318 (98.1%) | 760 (98.4%) | |
| Yes | 6 (1.9%) | 12 (1.6%) | |
| Had any other OIs (N=1,096) | | | 0.437 |
| No | 184 (56.8%) | 458 (59.3%) | |
| Yes | 140 (43.2%) | 314 (40.7%) | |
| Had any comorbidities (N=1,229) | | | 0.417 |
| No | 338 (92.1%) | 805 (93.4%) | |
| Yes | 29 (7.9%) | 57 (6.6%) | |
| Had any affective mental health disorder (N=1,229) | | | 0.001 |
| No | 320 (87.2%) | 801 (92.9%) | |
| Yes | 47 (12.8%) | 61 (7.1%) | |
| Time of ART initiation (N=1,157) | | | 0.794 |
| Same day | 155 (52.5%) | 441 (51.2%) | |
| First week (1-7 days) | 86 (29.2%) | 240 (27.8%) | |
| Second-fourth week | 38 (12.9%) | 125 (14.5%) | |
| After fourth week | 16 (5.4%) | 56 (6.5%) | |
| ART regimen (N=1,229) | | | 0.109 |
| EFV-based | 240 (65.4%) | 509 (59.0%) | |
| DTG-based | 121 (33.0%) | 334 (38.7%) | |
| Other | 6 (1.6%) | 19 (2.2%) | |
| Anyone knows your HIV status (N=1,073) | | | <0.001 |
| Yes | 187 (59.9%) | 551 (72.4%) | |
| No | 125 (40.1%) | 210 (27.6%) | |
| Spouse tested for HIV (N=977) | | | 0.038 |
| Yes | 72 (25.5%) | 234 (33.7%) | |
| No | 102 (36.2%) | 235 (33.8%) | |
| IDK | 108 (38.3%) | 226 (32.5%) | |
| Currently using khat stimulant (N=1,229) | | | 0.294 |
| No | 323 (88.0%) | 776 (90.0%) | |
| Yes | 44 (12.0%) | 86 (10.0%) | |
| Currently using any form of tobacco (N=1,229) | | | 0.604 |
| No | 352 (95.9%) | 832 (96.5%) | |
| Yes | 15 (4.1%) | 30 (3.5%) | |
| Currently drinking alcohol (N=1,175) | | | 0.710 |
| Yes | 130 (36.6%) | 291 (35.5%) | |
| No | 225 (63.4%) | 529 (64.5%) | |

*Had adherence assessment at 6- and/or 12-months.

**Abbreviations**: ART, antiretroviral; BMI, body mass index; DTG, Dolutegravir; EFV, Efavirenz; HF, health facility; HIV, human immunodeficiency virus; IDK, I don't know; IQR, interquartile range; OI, opportunistic infection; TB, tuberculosis.

**Sensitivity analysis**

Supplementary Table S3: Adherence to ART at 6- and 12-months follow-up among PLHIV in Ethiopia, March to June 2019

| | **6-months** | | **12-months** | |
|---|---|---|---|---|
| **Outcomes** | **Number** | **Percent** | **Number** | **Percent** |
| Adherence measurement* | (N=1,229) | | (N=1,229) | |
| Optimal | 1,165 | 94.8 | 1,169 | 95.1 |
| Suboptimal | 64 | 5.2 | 60 | 4.9 |

*Treating those who dropped out of the study as having optimal adherence.

Supplementary Table S4: Adherence to ART at 6- and 12-months follow-up among PLHIV in Ethiopia, March to June 2019

| | **6-months** | | **12-months** | |
|---|---|---|---|---|
| **Outcomes** | **Number** | **Percent** | **Number** | **Percent** |
| Adherence measurement* | (N=1,229) | | (N=1,229) | |
| Optimal | 737 | 60.0 | 698 | 56.8 |
| Suboptimal | 492 | 40.0 | 531 | 43.2 |

*Treating those who dropped out of the study as having suboptimal adherence.
